# Lyophilized aqueous extracts of *Mori Fructus* and *Mori Ramulus* protect Mesenchymal stem cells from •OH–treated damage: bioassay and antioxidant mechanism

**DOI:** 10.1186/s12906-017-1730-3

**Published:** 2017-05-02

**Authors:** Qian Jiang, Xican Li, Yage Tian, Qiaoqi Lin, Hong Xie, Wenbiao Lu, Yuguang Chi, Dongfeng Chen

**Affiliations:** 1 School of Chinese Herbal Medicine, Guangzhou University of Chinese Medicine, Waihuan East Road No.232, Guangzhou Higher Education Mega Center, Guangzhou, 510006 China; 2 Innovative Research & Development Laboratory of TCM, Guangzhou University of Chinese Medicine, Guangzhou, 510006 China; 3 School of Basic Medical Science, Guangzhou University of Chinese Medicine, Guangzhou, 510006 China; 4 The Research Center of Integrative Medicine, Guangzhou University of Chinese Medicine, Guangzhou, 510006 China

**Keywords:** Antioxidant, Electron transfer, Fe-chelating, Flavonoids, H^+^-transfer, Herbal medicine, Mesenchymal stem cells, *Mori Fructus*, *Mori Ramulus*

## Abstract

**Background:**

*Mori Fructus* and *Mori Ramulus* are two traditional Chinese herbal medicines from mulberries. The present work explores their beneficial effects on •OH–treated mesenchymal stem cells (MSCs) and discusses possible mechanisms.

**Methods:**

Lyophilized aqueous extracts of *Mori Fructus* (LAMF) and *Mori Ramulus* (LAMR) were prepared and analyzed using HPLC. LAMF and LAMR (along with morin) were further investigated for their effects on •OH-treated MSCs using the 3-(4,5-dimethylthiazol-2-yl)-2,5-diphenyl (MTT) assay. The direct antioxidation mechanisms were studied using 2-phenyl-4,4,5,5-tetramethylimidazoline-1-oxyl-3-oxide (PTIO•)-scavenging, 2,2′-azino-bis (3-ethylbenzo-thiazoline-6-sulfonic acid (ABTS^+^•)-scavenging and 1,1-diphenyl-2-picryl-hydrazl (DPPH•)-scavenging, as well as Cu^2+^-reducing and Fe^3+^-reducing antioxidant power. Finally, the indirect antioxidant mechanism was investigated based on the UV-vis spectra of Fe^2+^-chelation.

**Results:**

In each LAMF and LAMR, seven phytophenols were successfully measured by HPLC, including five flavonoids (morin, rutin, astragalin, isoquercitrin and luteolin) and two non-flavonoids (chlorogenic acid and maclurin). MTT assays revealed that LAMF, LAMR and morin could effectively increase the survival of •OH-treated MSCs at 10–100 μg/mL, and could effectively scavenge PTIO• (IC _50_ 6609.7 ± 756.6, 4286.9 ± 84.9 and 103.4 ± 0.9 μg/mL, respectively), DPPH• (IC _50_ 208.7 ± 3.0, 97.3 ± 3.1 and 8.2 ± 0.7 μg/mL, respectively) and ABTS^+^• (IC _50_ 73.5 ± 5.8, 34.4 ± 0.1 and 4.2 ± 0.2 μg/mL, respectively), and reduce Cu^2+^ (IC _50_ 212.5 ± 7.0, 123.2 ± 0.9 and 14.1 ± 0.04 μg/mL, respectively) & Fe^3+^ (IC _50_ 277.0 ± 3.1, 191.9 ± 5.2 and 5.0 ± 0.2 μg/mL, respectively). In the Fe^2+^-chelating assay, the five flavonoids produced much stronger shoulder-peaks than the two non-flavonoids within 420–850 nm.

**Conclusion:**

*Mori Fructus* and *Mori Ramulus,* can protect MSCs from •OH-induced damage. Such beneficial effects can mainly be attributed to the antioxidant action of phytophenols, which occurs via direct (ROS-scavenging) and indirect mechanism (Fe^2+^-chelating). The ROS-scavenging mechanism, however, include at least a H^+^-transfer and an electron-transfer (ET), and possibly includes a hydrogen-atom-transfer (HAT). In the Fe^2+^-chelating, flavonoids are more effective than non-flavonoids. This can be attributed to several adjacent planar chelating-sites between the 3-OH and 4-C = O, between the 4-C = O and 5-OH, or between the 3′-OH and 4′-OH in flavonoids. Such multiple-Fe^2+^-chelating reactions cause overlap in the UV-vis absorptions to deepen the complex color, enhance the peak strength, and form shoulder-peaks. By comparison, two non-flavonoids with catechol moiety produce only a weak single peak.

**Electronic supplementary material:**

The online version of this article (doi:10.1186/s12906-017-1730-3) contains supplementary material, which is available to authorized users.

## Background

As a type of multipotent stem cell, mesenchymal stem cells (MSCs) are expected to play a critical role in tissue repair, tissue regeneration and immune modulation [1, 2]. However, the longevity and functions of ex vivo culture-expanded MSCs are severely affected by oxidative stress. Excessive reactive oxygen species (ROS) inhibit MSC proliferation and osteogenic differentiation, lower MSC immunomodulation, and induce senescence and adipogenesis. This presents an obstacle for their clinical applications. Recently, Denu and co-workers suggested that further studies of oxidative stress in MSCs are required to accelerate the ex vivo expansion of MSCs and their in vivo engraftment, function and longevity [2].

**Fig. 1:**
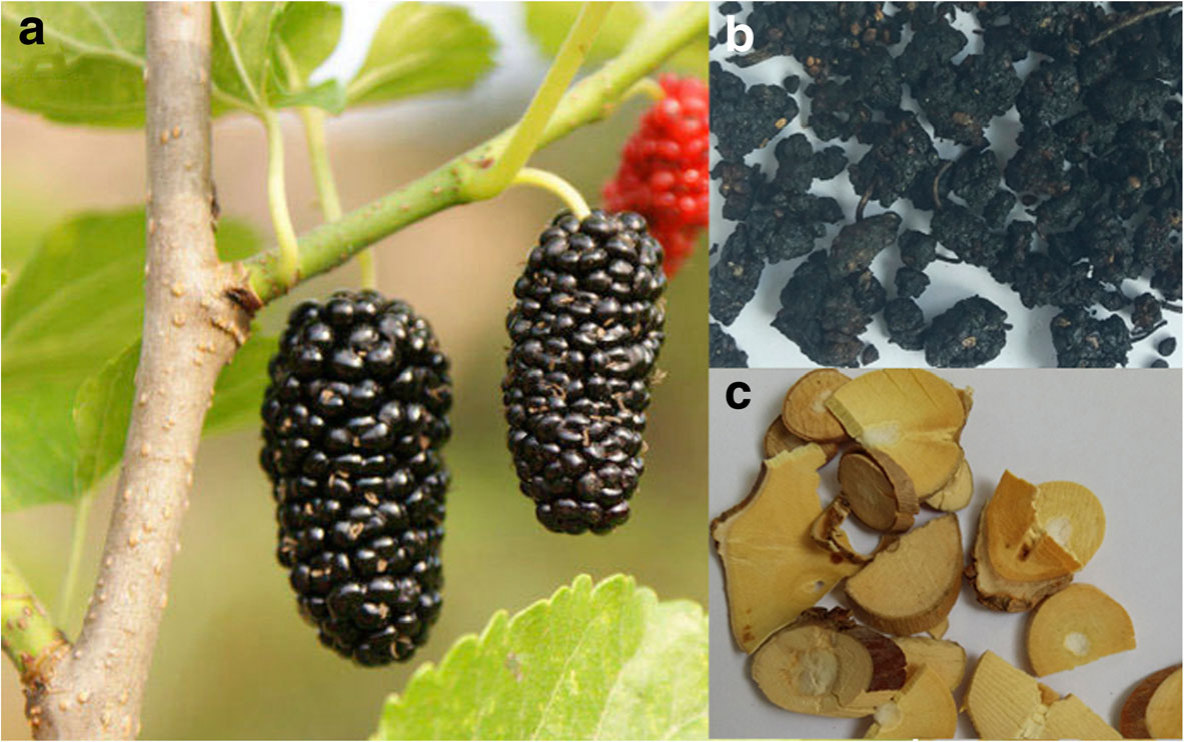
Images of *Morus alba L.* (**a**), *Mori Fructus (* **b**) and *Mori Ramulus (* **c**)

It may be a good choice to search for antioxidant nutrients from old-line plants with high regeneration capacity. It has been reported that outdoor plants are challenged by ROS-induced oxidative stress, not only from sunlight, air, water and heavy-metal outdoor pollution, but also by the plant itself which requires sufficient ROS for photosynthesis [3]. These old-line plants are thought to possess a powerful antioxidant system as defense against ROS-induced oxidative stress. Mulberry *(* *Morus alba L.* *Fig.* *1* *a)* is considered to be a typical example. According to the Oracle of Shang Dynasty (16th–11th, B.C.) in ancient China, *Morus alba L.* has survived for at least 3500 years. In China, *Morus alba L.* has been planted easily in various soils, even though they are frequently sheared or cut [4].

In the Tang Dynasty (618–906 CE), the dried fruit of *Morus alba L.* was recorded as a traditional Chinese herbal medicine in *Tang materia medica (Tang Ben Cao)* [5], a pharmacopoeia of the Tang Empire. In this ancient classic of traditional Chinese medicine (TCM), it is usually referred to as *Mori Fructus* (“Sangshen” in Chinese, Fig. 1b). From the perspectives of TCM, *Mori Fructus* can tonify *liver* and *kidney* and nourish *Yin* and *blood*. Thus, *Mori Fructus* is usually used to prepare some tonifying prescriptions such as Sang-Shen-Gao, which is now widely consumed in China [4]. Intake of *Mori Fructus* or its prescriptions can help one to recover from weakness. The tender twig of *Morus alba L.,* however, has also been used as a traditional Chinese herbal medicine for over 1000 years [6], and is referred to as *Mori Ramulus* (“Sangzhi” in Chinese. Fig. 1c) in China. *Mori Ramulus* is documented to dispel *wind* and to clear *heat-toxic*. The so-called *wind* and *heat-toxic* in TCM are from ROS-induced oxidative damage to lipids, tissues and cells, or to the immunologic and nervous systems [7].

From a chemical perspective, approximately 20 phytophenols with antioxidant potential have been successfully isolated from *Mori Fructus* or *Mori Ramulus*; these phytophenols embrace a number of flavonoids (e.g., morin), caffeoylquinic acids (e.g., chlorogenic acid), maclurin (a special phytophenol), etc. [8, 9]. The above-mentioned functions and chemical contents may partly be responsible for the fact that mulberries can feed silkworm larvae to imago. However, no studies have focused on their beneficial effects on MSCs.

The present study explores the effects of *Mori Fructus* and *Mori Ramulus* on •OH–treated MSCs*.* We believe that this study will help to understand the mechanisms of the beneficial effects of *Mori Fructus* and *Mori Ramulus* as Chinese Herbal medicines, as well as to provide additional information concerning the applications of natural phytophenols in cell transplantation engineering.

## Methods

### Materials and animals

*Mori Fructus* and *Mori Ramulus* were purchased from Kangmei Pharmaceutical Co. Ltd. (Shantou, China). Morin (C_15_H_10_O_7_, CAS 480–16-0, 98%), isoquercitrin (C_21_H_20_O_12_, CAS 482–35-9, 98%), astragalin (C_21_H_20_O_11_, CAS 480–10-4, 98%) and luteolin (C_15_H_10_O_6_, CAS 491–70-3, 98%) were purchased from Sichuan Weikeqi Biological Technology Co., Ltd. (Chengdu, China). Chlorogenic acid (C_16_H_18_O_9_, CAS 327–97-9, 98%) was purchased from the National Engineering Research Center of Traditional Chinese Medicine Solid State Manufacturing Technology (Nanchang, China). Rutin (C_27_H_30_O_16_, CAS 153–18-4, 98%) was purchased from Aladdin Industrial Corporation (Shanghai, China). Maclurin (C_13_H_10_O_6_, CAS 519–34-6, 98%) was obtained from BioBioPha (Kunming, China). Dulbecco’s modified Eagle’s medium (DMEM) and fetal bovine serum (FBS) were purchased from Gibco (Grand Island, NY, USA). CD44 was obtained from Wuhan Boster Co., Ltd. (Wuhan, China). DPPH• (1,1-diphenyl-2-picryl-hydrazl), neocuproine (2,9-dimethyl-1,10-phenanthroline), BHA (butylated hydroxyanisole), Trolox [(±)-6-hydroxyl-2,5,7,8-tetramethlychromane-2-carboxylic acid], 2,4,6-tripyridyl triazine (TPTZ), PTIO (2-phenyl-4,4,5,5-tetramethylimidazoline-1-oxyl-3-oxide) and neocuproine were obtained from Sigma-Aldrich Trading Co. (Shanghai, China). (NH_4_)_2_ABTS [2,2′-azino-bis (3-ethylbenzo-thiazoline-6-sulfonic acid diammonium salt)] was from Amresco Chemical Co. (Solon, OH, USA). Acetonitrile and water were of HPLC grade. All other reagents used in this study were purchased as the analytical grade from Guangzhou Chemical Reagent Factory (Guangzhou, China). Sprague-Dawley (SD) rats 4 weeks of age were obtained from the Animal Center of Guangzhou University of Chinese Medicine.

### Preparation of aqueous extracts of *Mori Fructus (*LAMF) *and Mori Ramulus* (LAMR)

**Fig. 2:**
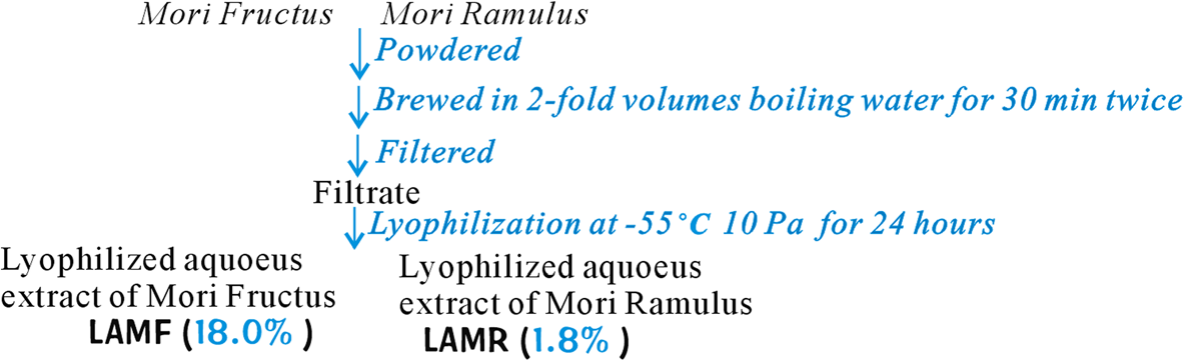
Preparation of lyophilized aqueous extracts of *Mori Fructus* (LAMF) and *Mori Ramulus* (LAMR)

*Mori Fructus* and *Mori Ramulus* were powdered and then extracted with distilled water. The aqueous extracts were lyophilized and stored at 4 °C for further analysis. The preparation procedure is detailed in Figs. 1 and 2.

### HPLC analysis of LAMF and LAMR

HPLC analysis was performed on a Waters e2695 system (Los Angeles, California, USA) equipped with an Agilent 5 TC-C_18_ column (250 mm × 4.6 mm, 5 μm) (Beijing, China). The mobile phase consisted of 0.2% phosphoric acid in water (A), 0.2% phosphoric acid in acetonitrile (B) (0–9 min, 15% B-20% B; 10–15 min, 20%B-25%B; 15–25 min, 25%B-30%B; 25–35 min, 30% B; 35–40 min, 30% B-35% B). The flow rate was 0.6 mL/min, the injection volume was 20 μL and the absorption was measured at 360 nm. In this study, chlorogenic acid, maclurin, rutin, isoquercitrin, astragalin, morin and luteolin in LAMF and LAMR were identified based on their retention times. They were quantified based on the corresponding standard curves generated using pure standards and their peak areas in the HPLC analysis of LAMF and LAMR.

### Protecting MSCs against oxidative stress-induced apoptosis (MTT assay)

The MSCs were cultured according to the method described in our previous report [10]. Briefly, bone marrow samples were obtained from the femurs and tibias of rats, and the resulting samples were diluted with DMEM (LG: low glucose) containing 10% FBS. The MSCs were obtained by gradient centrifugation at 900 × g for 30 min using a 1.073 g/mL Percoll system. The cells were then detached by treatment with 0.25% trypsin and passaged into culture flasks at a density of 1 × 10^4^ cells/cm^2^. The homogeneity of the MSCs was evaluated at passage 3 by flow cytometry based on their expression of CD44. These cells were then used for the following experiments.

The MSCs were seeded into 96-well plates (4 × 10^3^ cells/well). After adherence for 24 h, the cells were divided into three groups, including control, model and samples groups. The MSCs in the control group were incubated for 24 h in DMEM. The MSCs in the model group were injured for 1 h using FeCl_2_ (100 μM) followed by H_2_O_2_ (50 μM). The resulting mixture of FeCl_2_ and H_2_O_2_ was removed and the MSCs were incubated for 24 h in DMEM. The MSCs in the samples groups were injured and incubated for 24 h in DMEM in the presence of various concentrations of samples. After the incubation, the cells were treated with 20 μL of MTT (5 mg/mL in PBS), and the resulting mixtures were incubated for 4 h. The culture medium was subsequently discarded and replaced with 150 μL of DMSO. The absorbance of each well was then measured at 490 nm using a Bio-Kinetics plate reader (PE-1420; Bio-Kinetics Corporation, Sioux Center, IA, USA). The serum medium was used for the control group and each sample test was repeated in five independent wells.

### Free radical-scavenging assays in vitro

The assays include PTIO•-scavenging, DPPH•-scavenging and ABTS^+^•-scavenging assays. In the PTIO•-scavenging assay, 80 μL of an aqueous PTIO• solution (0.1 mM) was mixed with 20 μL of an aqueous or alcoholic solution of sample at various concentrations. The mixture was maintained at 37 °C for 2 h, and the absorbance at 560 nm was then measured using a microplate reader (Multiskan FC, Thermo scientific, Shanghai, China). The PTIO• inhibition percentage was calculated as:

$$ \mathrm{Inhibition}\%=\frac{{\mathrm{A}}_0\hbox{-} \mathrm{A}}{{\mathrm{A}}_0}\times 100\% $$

where A_0_ is the absorbance of the control without sample and A is the absorbance of the reaction mixture with sample.

The DPPH•-scavenging and ABTS^+^•-scavenging assays were based on previous reports [11, 12]. In the DPPH•-scavenging assay, 0.9 mL of an ethanolic solution of DPPH• (0.1 mM) was mixed with 0.6 mL of an ethanolic or aqueous solution of sample at various concentrations. The mixture was maintained at room temperature for 30 min and the absorbance at 519 nm was then measured. In the ABTS^+^•-scavenging assay, the ABTS^+^• was produced by mixing 200 μL of (NH_4_)_2_ABTS (7.4 mM) with 200 μL of K_2_S_2_O_8_ (2.6 mM). After incubation in the dark for 12 h, the mixture was diluted with methanol (approximately 1:50) so that the absorbance at 734 nm was 0.70 ± 0.02. Then, the diluted ABTS^+^• solution (800 μL) was added to 200 μL of an ethanolic or aqueous solution of sample at various concentrations and then mixed thoroughly. After the reaction mixture stood for 6 min, the absorbance at 734 nm was measured using a spectrophotometer. The percentage inhibition of DPPH•-scavenging or ABTS^+^•-scavenging was calculated using the formula described above.

### Metal-reducing power assays

Metal-reducing power assays include the ferric reducing antioxidant power (FRAP) assay and the cupric (Cu^2+^) reducing capacity. The FRAP assay was adapted from Benzie and Strain [13]. Briefly, the FRAP reagent was prepared freshly by mixing 10 mM TPTZ, 20 mM FeCl_3_ and 0.25 M acetate buffer at 1:1:10 at pH 3.6. The test sample (*x* = 20–100 μL, 0.5 mg/mL) was added to(100- *x*)μL of 95% ethanol followed by 400 μL of FRAP reagent. The absorbance was measured at 593 nm after a 30 min incubation at ambient temperatures using distilled water as the blank. The relative reducing power of the sample as compared with the maximum absorbance, was calculated by the following formula:

$$ Relative\  reducing\  effect\%=\frac{A-{A}_{\min }}{A_{\max }-{A}_{\min }}\times 100\% $$

where A_min_ is the absorbance of the control without sample, A is the absorbance of the reaction mixture with sample, and A_max_ is the greatest absorbance of the reaction mixture with sample. The cupric (Cu^2+^) reducing capacity was determined as previously described [14]. Briefly, 125 μL of an aqueous solution of CuSO_4_ (10 mM), 125 μL of an ethanolic solution of neocuproine (7.5 mM), and (750-*x*) μL of CH_3_COONH_4_ buffer solution (100 mM, pH 7.5) were added to test tubes containing different volumes of samples (1 mg/mL, *x* = 20–120 μL). Then, the total volume was adjusted to 1000 μL with the buffer and the solution was mixed vigorously. The absorbance at 450 nm was measured against a buffer blank after 30 min (Unico 2100, Shanghai, China). The relative reducing power of the sample as compared with the maximum absorbance was calculated using the above formula.

### Ultraviolet (UV) spectra determination of Fe^2+^-chelating

Ultraviolet (UV) spectra of Fe^2+^-chelating of LAMF, LAMR, chlorogenic acid, maclurin, morin, rutin, astragalin and luteolin were determined according to a previously described method [15]. Briefly, 300 μL of an aqueous solution of LAMF, LAMR, or relevant phytophenols was added to 700 μL of an aqueous solution of FeCl_2_•4H_2_O (10 mg/mL). The total volume was adjusted to 1000 μL and the solution was then mixed vigorously. The resulting mixture was incubated at room temperature for 24 h. The product mixtures were then imaged using a smartphone (Samsung, Galaxy A7, China). Subsequently, the supernatant was collected and a spectrum was obtained using a UV/Vis spectrophotometer (Jinhua 754 PC, Shanghai, China) from 200 to 1000 nm.

### Statistical analysis

The IC_50_ values were calculated by linear regression analysis. All linear regressions in this paper were analyzed using Origin 6.0 professional software. The determination of significant differences between the mean IC_50_ values of the sample and positive controls was performed using one-way ANOVA and t-test. The analysis was performed using SPSS software 18.0 (SPSS Inc., Chicago, IL) for windows. *P* < 0.05 was considered to be statistically significant.

## Results and discussion

In TCM, Chinese herbal medicines are always prepared as a water decoction for clinical applications. Considering the relevance to TCM and cellular physiological aqueous environments, two Chinese herbal medicines, *Mori Fructus* and *Mori Ramulus,* were also decocted with boiling water to obtain their aqueous extracts. To avoid destroying the antioxidant nutrients, two aqueous extracts were lyophilized at −55 °C under a vacuum condition (10 Pa). The yields were calculated as 18.0% and 1.8%, respectively (Fig. 2).

**Fig. 3:**
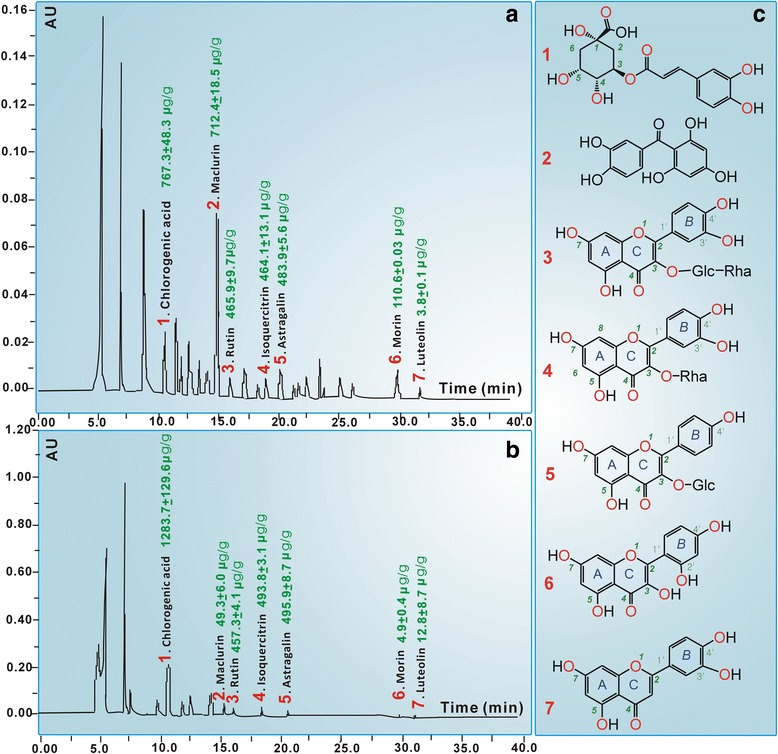
HPLC profiles and chemical contents of LAMA (**a**) and LAMR (**b**), and structures of relevant phytophenols (**c**)

To obtain chemical information on LAMF and LAMR, an HPLC analysis was conducted in the study. The results revealed that there were at least seven phytophenols, including five flavonoids (morin, rutin, quercetin, isoquercitrin and luteolin) and two non-flavonoids (chlorogenic acid and maclurin). Among them, maclurin and morin can be found only in the Moraceae family, and they are regarded as marker components of the Moraceae family [16–18]. However, maclurin has been evaluated in our previous study [18]. Thus, morin was evaluated for its protective effects in this study, along with two extracts, LAMF and LAMR. As seen in Fig. 3, LAMF, LAMR and morin dose-dependently increased the protective percentages. However, the tested concentrations of morin were much lower than those of LAMF or LAMR. The other phytophenols, such as chlorogenic acid, maclurin, rutin, isoquercitrin and luteolin, have been reported to protect cells similarly from oxidative damage by our and other laboratories [15, 18, 21–23]. This suggests that phytophenols can be considered as bioactive compounds for the protective effects of LAMF and LAMR, especially morin and maclurin, which can be considered as bioactive and marker compounds. This can also be partly supported by the evidence that morin has been observed in ethanolic extracts of *Mori Ramulus* (mulberry twigs) [19], and high glucose induced oxidative stress-mediated apoptosis in primary rat hepatocytes [20]. These findings, that LAMF, LAMR and relevant phytophenols can release the oxidative stress of MSCs, can also explain the latest findings that mulberries have the potential to decelerate osteoporosis that is associated with oxidative stress in an experimental ovariectomic rat model [24].

Our previous study reported that such protective effects were due to the ROS-scavenging (particularly •OH-scavenging) of phytophenols [25]. To test this possibility, we evaluated their ROS-scavenging levels. However, typical ROS forms are transient and have very short half-lives, e.g., •OH radical (10^−9^ s), •O_2_^−^ radical **(**10^−6^ s), and lipid-peroxide radicals (LOO•, 10^−2^ s). These radicals cannot be detected directly using standard analytical technologies (e.g., spectrophotometry, HPLC, and fluorimetry). Thus, the stable oxygen-centered radical, PTIO•, was selected as the ROS mimic for the investigation. In the PTIO• radical, the unpaired electron is associated with the O atom, and thus, it is an oxygen-centered radical; the amine oxide zwitterion moiety renders it a hydrophilic species. The levels of PTIO•-scavenging can well reflect those of ROS-scavenging in aqueous solution.

As shown in Additional file 1 and Table 1, LAMF, LAMR, morin and chlorogenic acid all exhibited good dose-dependent responses in the PTIO-scavenging assay. The results are consistent with those from nitrogen-centered radical scavenging assays (DPPH•-, and ABTS^+^•-scavenging assays) [26], and those from metal-reducing power assays (FRAP and Cu^2+^-reducing) (Additional file 1). These results indicated that LAMF and LAMR (especially phytophenols) could directly scavenge ROS, and that ROS-scavenging was hence considered as a direct antioxidant mechanism.

**Table 1:** The IC_50_ values of LAMF, LAMR, morin, and chlorogenic acid in various antioxidant assays (μg/mL or μM)

Assays	LAMF	LAMR	morin	chlorogenic acid	luteolin	Trolox
PTIO• Scavenging	6609.7 ± 756.6^d^	4286.9 ± 84.9^c^	103.4 ± 0.9^b^ (344.6 ± 2.9^C^)	99.6 ± 2.3^b^ (281.4 ± 6.5^B^)	ND	40.6 ± 6.1^a^ (162.2 ± 24.2 ^A^)
DPPH•-scavenging	208.7 ± 3.0^d^	97.3 ± 3.1^c^	8.2 ± 0.7^b^ (27.1 ± 2.2 ^D^)	5.6 ± 0.01^a^ (15.8 ± 0.03^B^)	3.2 ± 0.1^a^ (11.1 ± 0.2^A^)	4.5 ± 0.1^a^ (18.1 ± 0.4 ^C^)
ABTS• scavenging	73.5 ± 5.8^c^	34.4 ± 0.1^b^	4.2 ± 0.2^a^ (13.9 ± 0.6 ^B^)	5.8 ± 0.3^a^ (16.4 ± 0.9 ^B^)	2.6 ± 0.1^a^ (9.0 ± 0.2^A^)	1.9 ± 0.6^a^ (7.7 ± 2.6^A^)
FRAP	277.0 ± 3.1^c^	191.9 ± 5.2^b^	5.0 ± 0.2^a^ (16.7 ± 0.8 ^B^)	4.9 ± 1.0^a^ (13.9 ± 2.7^A^)	5.8 ± 0.1^a^ (20.1 ± 0.2^C^)	7.0 ± 0.4^a^ (28.1 ± 1.7 ^D^)
Cu^2+^-reducing	212.5 ± 7.0^c^	123.2 ± 0.9^b^	14.1 ± 0.04^a^ (46.5 ± 0.1 ^B^)	12.5 ± 0.6^a^ (35.2 ± 1.6 ^A^)	10.2 ± 0.3^a^ (36.8 ± 0.9^A^)	15.7 ± 0.6^a^ (62.7 ± 2.5 ^C^)

The IC_50_ value is defined as the concentration for a 50% effect and is expressed as the mean ± SD (*n* = 3). The IC_50_ values without parentheses are in μg/mL units; The IC_50_ values in parentheses are in μM units. Mean IC_50_ values in μg/mL units with different superscripts (a, b) in the same row are significantly different (*p* < 0.05), while those with the same superscripts are not significantly different (*p* < 0.05). Mean IC_50_ values in μM units with different superscripts (A, B) in the same row are significantly different (*p* < 0.05), while those with the same superscripts are not significantly different (*p* < 0.05). FRAP ferric ion reducing antioxidant power; ABTS, 2,2′-azino-bis (3-ethylbenzo-thiazoline-6-sulfonic acid; DPPH, 1,1-diphenyl-2-picryl-hydrazl; PTIO, 2-phenyl-4,4,5,5-tetramethylimidazoline-1-oxyl 3-oxide; LAMF, lyophilized aqueous extract of *Mori Fructus*; lyophilized aqueous extract of *Mori Ramulus*. Trolox acted as the positive control. The dose-response curves are listed in Additional file 1

Nevertheless, the above-mentioned assays are actually based on different mechanisms. PTIO• scavenging is reported as an H^+^-transfer pathway [27]. FRAP, a reaction under acidic conditions (pH 3.6) is considered as an electron-transfer (ET) process, because the acidic solution suppresses the ionization of phytophenols [28, 29]. The fact that LAMF and LAMR, along with relevant phytophenols, have inhibitory effects on PTIO• and reductive effects on Fe^3+^, implies that their ROS-scavenging action may include H^+^-transfer and ET pathways (Table 1) [15, 18, 21–23]. Our assumption is further supported by previous evidence concerning chlorogenic acid [30], maclurin [18], flavonoids [31], morin [31, 32], astragalin [29], morin [32], rutin and isoquercitrin [15, 33]. In short, direct antioxidation (ROS-scavenging) may take place in the protection of •OH-induced MSCs by LAMF and LAMR, and the direct mechanism at least includes a H^+^-transfer and an ET, and possibly includes a HAT.

According to the IC_50_ values in Table 1 and the previous literature [15, 18, 21–23], phytophenols (flavonoids and non-flavonoids) always exhibited greater ability than two extracts (LAMF and LAMR,). This indicates that phytophenols may be the main bioactive components of the protective and antioxidant effects of LAMF and LAMR. In fact, some phytophenols have been recently manufactured as novel antioxidant materials (e.g., morin as nanomaterial [34]).

**Fig. 4:**
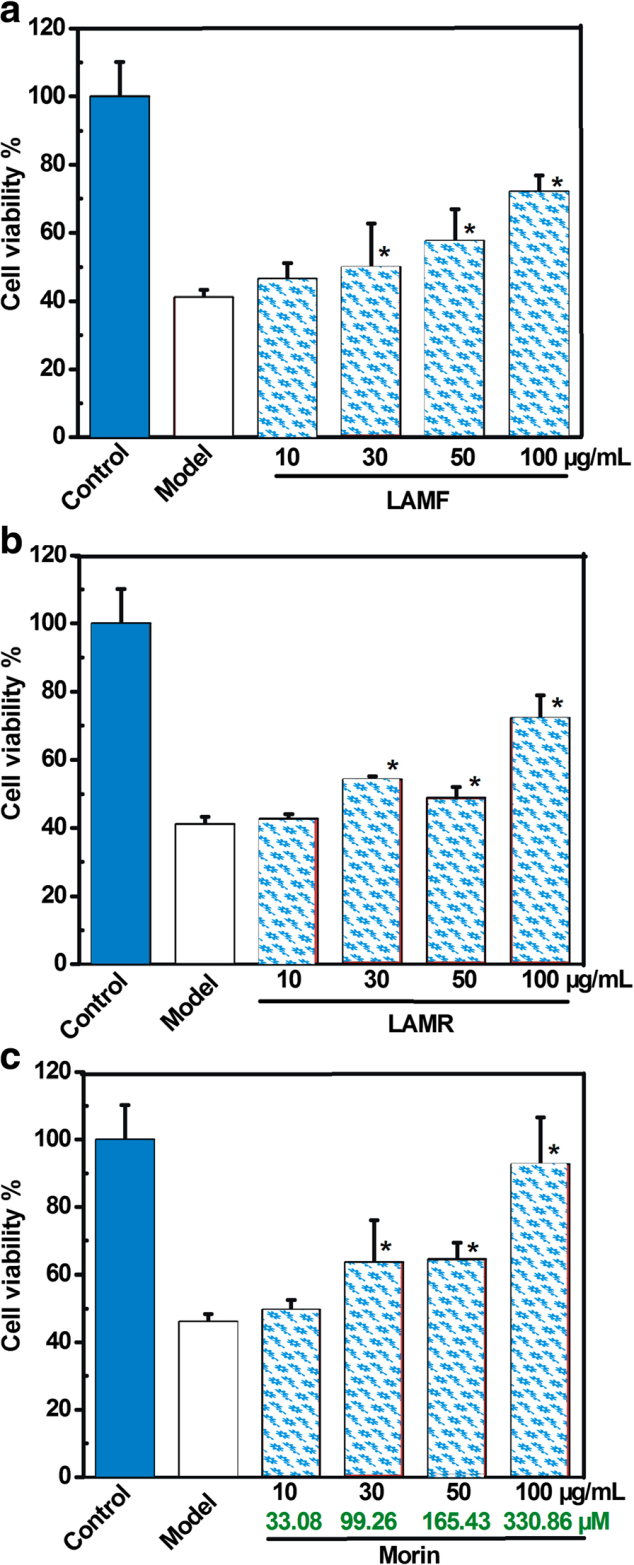
LAMF (**a**), LAMR (**b**), and morin (**c**) protect MSCs against •OH-induced apoptosis. Cell viability was assessed using the MTT method. Experiments were performed with 3 different batches of cells, and each batch was tested in triplicate. Data are the mean ± SD values. (*) *p* < 0. 05, compared with MSCs damage following FeCl_2_ *plus* H_2_O_2_. LAMF, Lyophilized aqueous extract of *Mori Fructus*; LAMR, lyophilized aqueous extract of *Mori Ramulus*

**Fig. 5:**
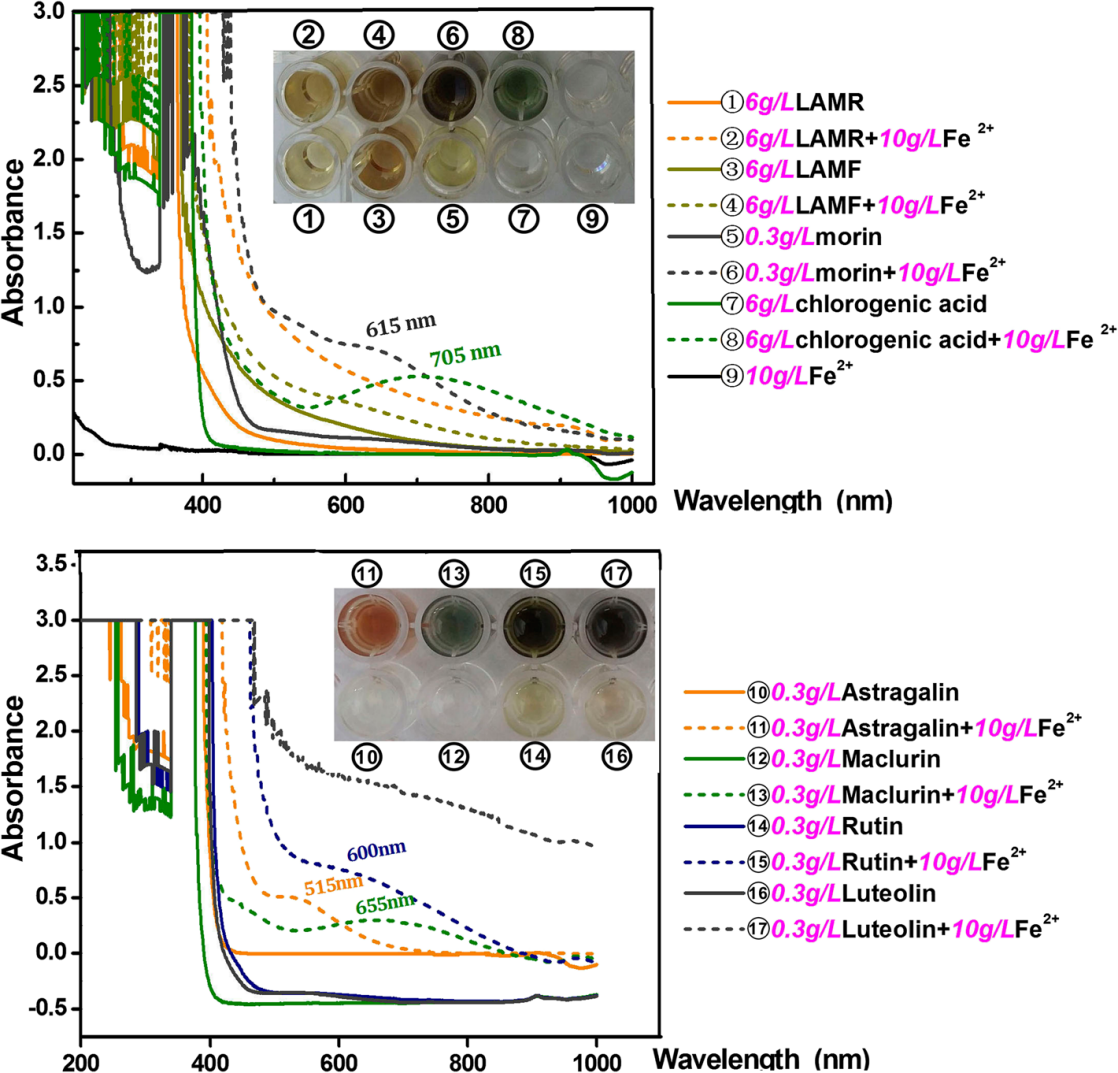
UV spectra of chelation products of LAMF, LAMR and relevant phytophenols with excess Fe^2+^ (The inset figures are the appearances of chelation products. The Fe^2+^-chelating ability of isoquercitrin was reported in our previous paper [15])

However, the generation of •OH in cells is known to rely on Fe^2+^ as a catalyst. A typical example is the Fenton reaction (Fe^2+^ + H_2_O_2_ → Fe^3+^+ •OH + •OH^−^). Thus, decreasing the levels of Fe^2+^ via a chelating effect is considered to be an indirect antioxidant mechanism for scavenging •OH radicals. In fact, iron-chelation now has been developed as a new therapy for some oxidative stressed diseases [35]. In the present study, all relevant phytophenols exhibited higher Fe^2+^-chelating abilities than LAMF and LAMR (Figs. 4 and 5). This clearly indicates that Fe^2+^-chelating may be one pathway for the antioxidant action of LAMF and LAMR, and further supports the assumption that phytophenols may be the bioactive antioxidant compounds of LAMF and LAMR.

However, the results in Fig. 5 indicate that morin produced a much greater response at a lower concentration (0.3 mg/mL, 1.0 mmol/L) than chlorogenic acid did at a higher concentration (6 mg/mL, 16.9 mmol/L). This can be explained by their molecular conformation.

**Fig. 6:**
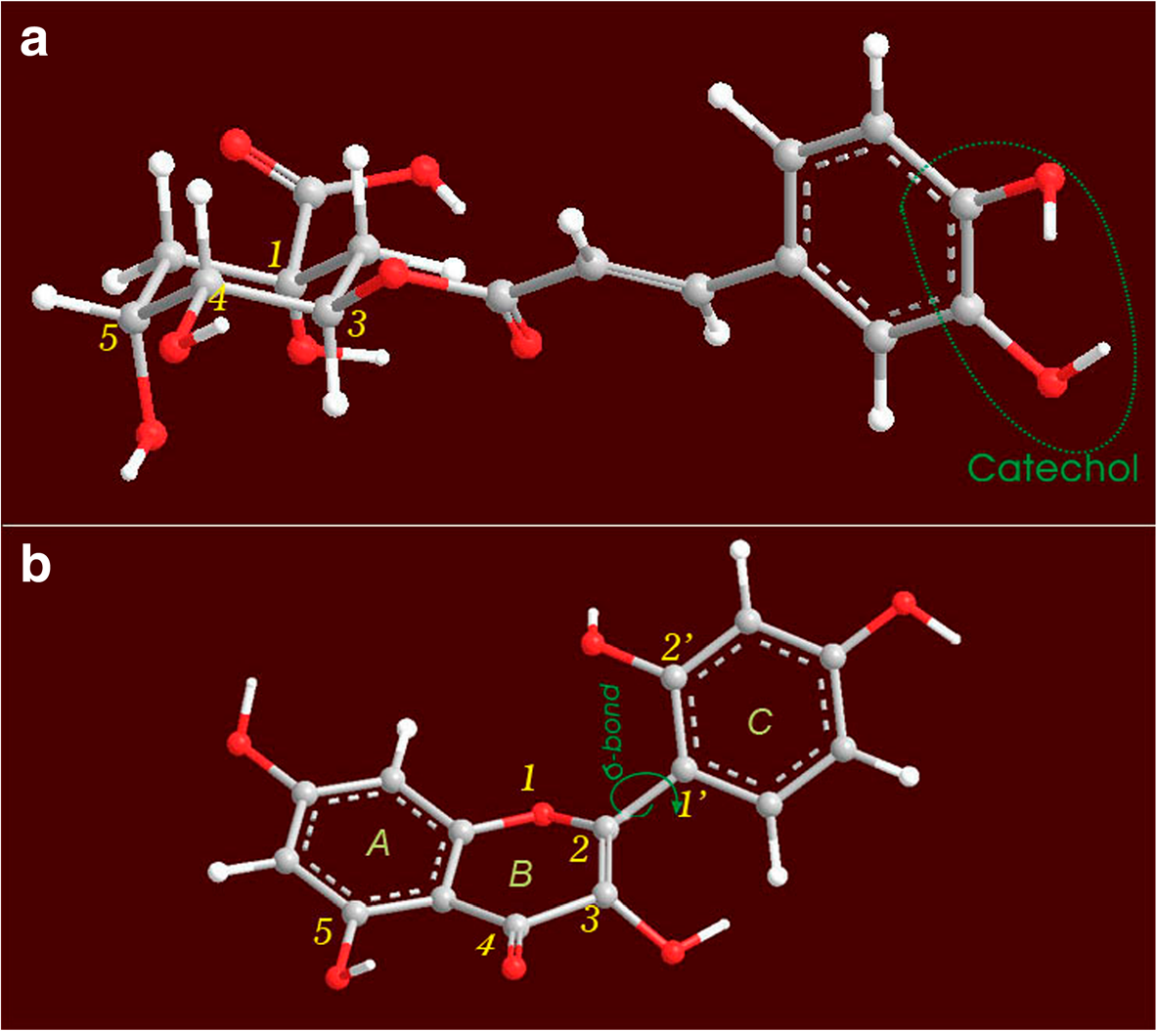
The preferential conformation-based ball-stick models of chlorogenic acid (**a**) and morin (**b**). (The models were created by ChemOffice 2004, CambridgeSoft)

As illustrated in Fig. 6a, chlorogenic acid has only one site for a metal chelating reaction, i.e., a catechol moiety attached to a benzene ring. The adjacent 4,5-dihydroxyl groups in the quinic acid ring cannot chelate metals to form a stable ringed complex because they are not in a planar conformation: 4-OH is in an axial-bond (*a*-bond) and 5-OH is in an equatorial-bond (*e*-bond). A similar situation is found to exist in the maclurin molecule [18]. Hence, maclurin also has only one chelating-site.

In flavonoid molecules, the situation is quite different. For example, in the morin molecule, there are two chelating-sites with planar conformations (Fig. 6b), i.e., between 3-OH and 4-C = O and between 4-C = O and 5-OH. Based on the above discussion and on previous literatures [36, 37], we propose the reaction of Fe-chelation with chlorogenic acid and morin as follows:

It is worth mentioning that the site between 1-O and 2′-OH in morin (Fig. 6b) is actually non-planar because the σ-bond between 2-C and 1′-C can rotate freely and there is often a dihedral angle between the B ring and the C ring [38]. Thus the site between 1-O and 2′-OH cannot readily form a ringed complex of metal chelation.

**Fig. 7:**
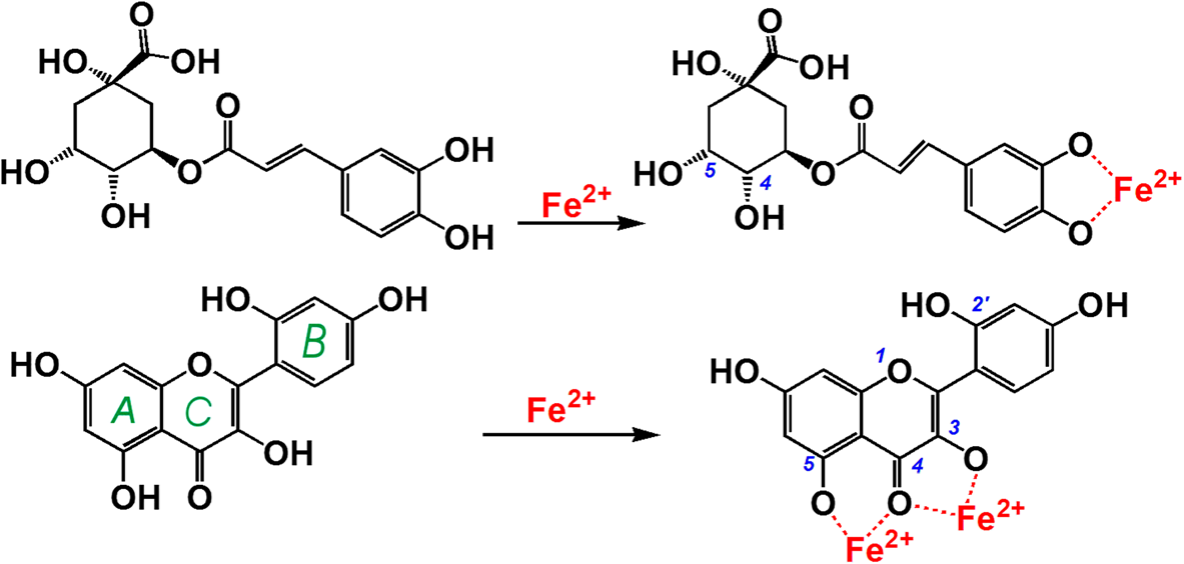
The proposed reaction of Fe^2+^-chelating with chlorogenic acid (**a**), and with morin (**b**)

The chelating reactions in Fig. 7 and in previous work [15] can also be used to explain the shape of the peaks that are obtained in UV spectra. Chlorogenic acid with one chelating-site gave rise to a symmetrical single peak, while morin yielded a broad shoulder peak. The broad shoulder peak was speculated to be a superposition of multi-peaks from multiple chelating-sites. Similar Fe^2+^-chelating peaks were also observed with other phytophenols, such as astragalin, rutin, luteolin (Fig. 5), and isoquercitrin [15], because these flavonoids contain multi-chelating-sites, such as between 4-C = O and 5-OH and between 3-OH and 4-C = O in astragalin; between 3-OH and 4-C = O, between 4-C = O and 5-OH, and between 3′-OH and 4′-OH in rutin and isoquercitrin; between 4-C = O and 5-OH and between 3′-OH and 4′-OH in luteolin (Fig. 3c).

## Conclusion

As two Chinese herbal medicines from mulberry, *Mori Fructus* and *Mori Ramulus* contain at least seven phytophenols including five flavonoids (morin, rutin, astragalin, isoquercitrin and luteolin) and two non-flavonoids (chlorogenic acid and maclurin) that can increase the survival of •OH-treated MSCs. Such beneficial effects can mainly be attributed to a phenolic antioxidant action that is fulfilled via a direct mechanism (ROS-scavenging) and an indirect mechanism (Fe^2+^-chelating). The ROS-scavenging pathway, however, includes at least a H^+^-transfer pathway and an ET pathway, and possibly includes a HAT pathway. In the Fe^2+^-chelating pathway, five flavonoids exhibited much greater Fe^2+^-chelating ability than non-flavonoids. This can be attributed to several chelating-sites with adjacent planar moieties between 3-OH and 4-C = O, between 4-C = O and 5-OH, or between 3′-OH and 4′-OH in these flavonoids. Such multiple chelation reactions create an overlap in the UV-vis absorptions to deepen the complex color, enhance the strength of the peak and form shoulder peaks in the spectra. By comparison, the non-flavonoids chlorogenic acid and maclurin with a catechol moiety produce only a very weak single peak. These findings will help to understand the nutritive or medicinal effect of *Mori Fructus* and *Mori Ramulus* and will also provide new information regarding natural products in stem cell transplantation engineering.

## Additional file

Additional file 1: The dose response curves. (PNG 233 kb)

## Abbreviations

ABTS: [2, 2′-azino-bis (3-ethylbenzo-thiazoline-6-sulfonic acid diammonium salt)]
DMEM: Dulbecco’s modified Eagle’s medium
DPPH•: (1, 1-diphenyl-2-picryl-hydrazl)
ET: Electron transfer
FBS: Fetal bovine serum
FRAP: ferric reducing antioxidant power
HAT: Hydrogen atom transfer
LAMF: Aqueous extract of *Mori Fructus*
LAMR: Aqueous extract of *Mori Ramulus*
MSCs: Mesenchymal stem cells
MTT: [3- (4, 5-dimethylthiazol-2-yl)-2, 5-diphenyl]
PTIO: 2-phenyl-4, 4, 5, 5-tetramethylimidazoline-1-oxyl-3-oxide
ROS: Reactive oxygen species
SD: standard deviation
TCM: Tradition Chinese Medicine
TPTZ: 2, 4, 6-tripyridyl triazine
Trolox: [(±)-6-hydroxyl-2, 5, 7, 8-tetramethlychromane-2-carboxylic acid]
