# Supplementary figures and images for: Lyophilized aqueous extracts of Mori Fructus and Mori Ramulus protect Mesenchymal stem cells from •OH–treated damage: bioassay and antioxidant mechanism
Source: BMC Complement Altern Med. 2017 May 2;17:242. doi: 10.1186/s12906-017-1730-3 (PMC5414230; doi:10.1186/s12906-017-1730-3)

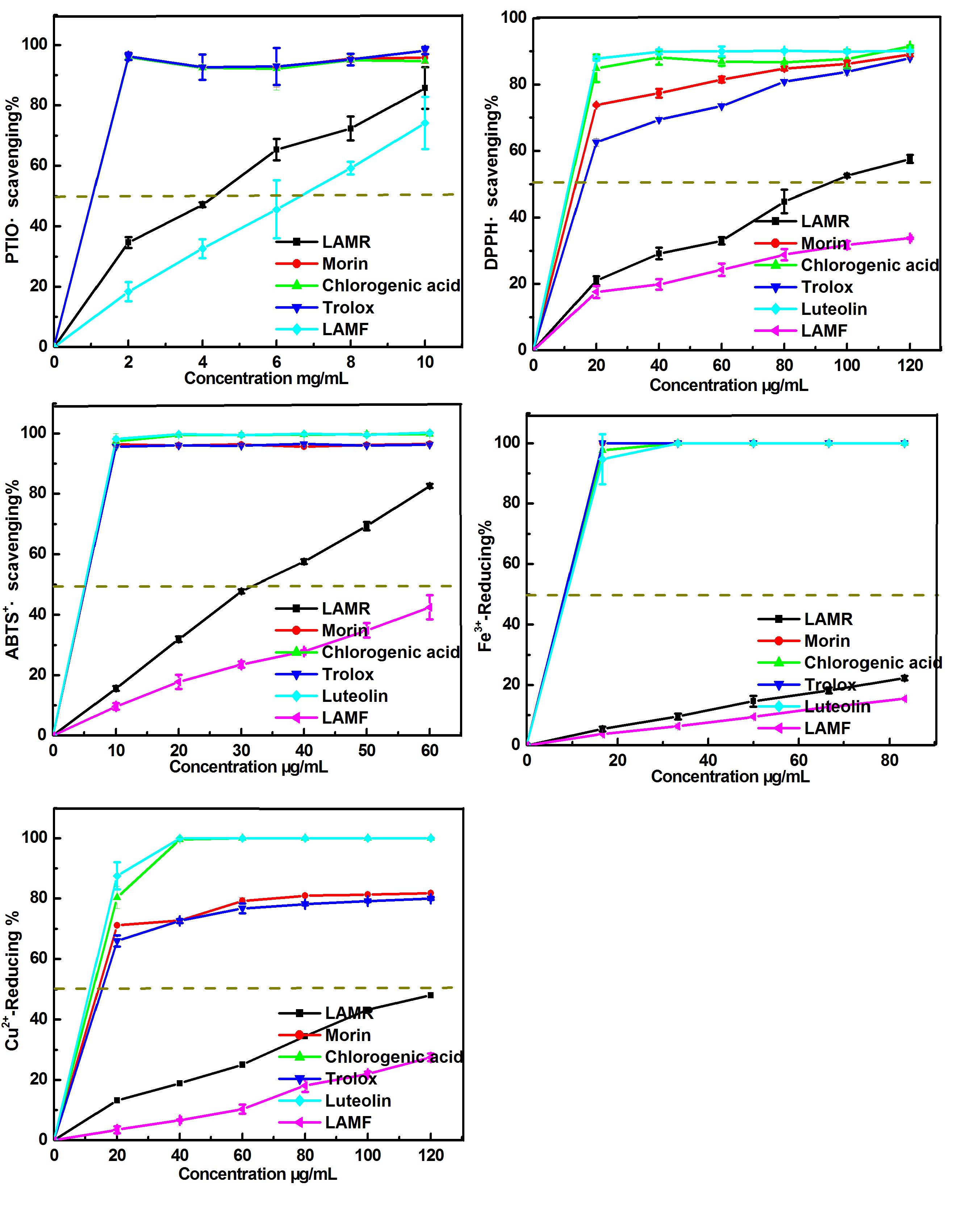

Supplement: Additional file 1: — The dose response curves. (PNG 233 kb) [file 12906_2017_1730_MOESM1_ESM.png]
